# Antimalarial combination therapies increase gastric ulcers through an imbalance of basic antioxidative-oxidative enzymes in male Wistar rats

**DOI:** 10.1186/s13104-020-05073-7

**Published:** 2020-04-23

**Authors:** Muhamudu Kalange, Miriam Nansunga, Keneth Iceland Kasozi, Josephine Kasolo, Jackline Namulema, Jovile Kasande Atusiimirwe, Emanuel Tiyo Ayikobua, Fred Ssempijja, Edson Ireeta Munanura, Kevin Matama, Ibrahim Semuyaba, Gerald Zirintunda, Alfred Omachonu Okpanachi

**Affiliations:** 1 Department of Physiology, Faculty of Biomedical Sciences, Kampala International University, Western Campus, Box 71, Bushenyi, Uganda; 2 Department of Physiology, College of Health Sciences, Makerere University, Box 7062, Kampala, Uganda; 3 Department of Physiology, Faculty of Biomedical Sciences, School of Medicine, Soroti University, Soroti, Uganda; 4 Department of Anatomy, Faculty of Biomedical Sciences, Kampala International University, Western Campus, Box 71, Bushenyi, Uganda; 5 Department of Pharmacy, College of Health Sciences, Makerere University, Box 7062, Kampala, Uganda; 6 Department of Therapeutics and Toxicology, School of Pharmacy, Kampala International University Western Campus, Box 71, Bushenyi, Uganda; 7 Department of Animal Production, Faculty of Agriculture and Animal Sciences, Busitema University Arapai Campus, Box 203, Soroti, Uganda; 8 Department of Physiology, Faculty of Medicine, Uzima University College CUEA, Box 2502, Kisumu, Kenya; 9 Department of Physiology, Faculty of Health Sciences, Busitema University, Mbale, Uganda

**Keywords:** Antimalarials, Pharmacodynamics of antimalarial agents, Malaria in developing countries, Gastric ulcers

## Abstract

**Objective:**

Antimalarials are globally used against plasmodium infections, however, information on the safety of new antimalarial combination therapies on the gastric mucosa is scarce. The aim of this study was to investigate the effects of Artesunate-Amodiaquine and Artemether-Lumefantrine on ulcer induction. Malondialdehyde (MDA), reduced glutathione (GSH) and major histological changes in male Wistar rats following ulcer induction using Indomethacin were investigated. Gastric ulcers were in four groups; Group I was administered Artesunate, group II received Artesunate-Amodiaquine, group III received Artemether-Lumefantrine, and group IV was a positive control (normal saline). Group V was the negative control consisting of healthy rats.

**Results:**

Antimalarial combination therapies were associated with a high gastric ulcer index than a single antimalarial agent, Artesunate. In addition, levels of MDA were significantly higher in the combination of therapies while levels of GSH were lower in comparison to Artesunate and the negative control. Microscopically, antimalarial combination therapies were associated with severe inflammation and tissue damage than Artesunate in the gastric mucosa showing that antimalarial combination therapies exert their toxic effects through oxidative stress mechanisms, and this leads to cellular damage. Findings in this study demonstrate a need to revisit information on the pharmacodynamics of major circulating antimalarial agents in developing countries.

## Introduction

Antimalarial single therapies (AMTs) are the aminoquinoline and artemisinin derivatives and artemisinin-based combination therapies and the development of resistance against them is a major public health threat especially in endemically infected countries with malaria parasites [1, 2]. The aminoquinoline derivatives (including quinine, chloroquine, amodiaquine, naphthoquinone, piperaquine, and mefloquine), are the prototype AMTs that have been used admist reports of varying adverse effects including toxicity concerns and increasing frequency for the development of drug resistance [3, 4]. These aminoquinolines are also aggressive to the gastric mucosa precipitating gastric ulceration [3, 5]. The derivatives of artemisinin (including artesunate, dihydroartemisinin, and artemether), are generally safer with limited side effects [6]. Their efficacy against malaria parasites is however lower due to their lower half-life compared to that of aminoquinolines [7]. The artemisinins have been shown to be safe on the gastric mucosal integrity [8, 9]. The artemisinin-based antimalarial combination therapies are recommended as the first-line treatment for uncomplicated malaria and this has been widely adopted [10]. This therapy involves a combination of artemisinin and aminoquinoline derivatives into a single oral treatment. The combination of tehse two drugs ideally presents different safety challenges compared to the individual drugs comprised therein [11]. In Africa, the use of antimalarial combinations such as Artemisinins is common for the management of malaria [12, 13], demonstrating their importance in developing countries.

The gastric mucosa is the inner protective lining of the gastric wall, made of an adherent mucus-bicarbonate-prostaglandin layer on a glandular epithelium [14]. Its integrity and efficiency depend on the thickness of the mucus layer, continuity of glandular epithelium, adequate circulation and anti-oxidative activity of gastric tissue [15]. The gastric mucosa is continuously exposed to endogenous and exogenous factors with protective or damaging effects [15, 16]. Drugs including antimalarials are among the exogenous substances known to affect the gastric mucosa through oxidative stress mechanisms [12, 13, 17]. Reactive oxygen species lead to lipid peroxidation through increased levels of malondialdehyde (MDA) and this disrupts the integrity of cell membranes leading to mucosal ulceration [18–22]. The glutathione system is an antioxidative system in the cell which prevents the accumulation of reactive oxygen species [23–26], thus hindrances to the functioning of the antioxidant system lead to increased tissue pathology. Lumefantrine, known to increase tissue oxidative stress [27, 28], has been incorporated with artemether into a combination therapy for the treatment of malaria [29–32]. However, information on the effects of this combination therapy on gastric ulcers remains to be established. The use of artesunate-amodiaquine has been associated with gastrointestinal complications like vomiting, diarrhea and abdominal pain [33, 34]. The objective of the study was to establish the gastric mucosal effects in Wistar rats of the common antimalarials used in developing countries.

## Main text

### Methods

#### Study design

This was an experimental study in which 25 adult male Wistar rats kept at Kampala International University Western Campus were assigned random numbers for experimental grouping as described previously [35]. Animals were exposed to good husbandry practices through access to sufficient quality food and water adlibitum, exposure to daylight 12 h and sufficient spacing to minimise stress as previously described [35]. Gastric ulcers were induced in only four experimental groups using indomethacin [36]. Rats were fasted for about 24 h, and then orally treated with indomethacin at 40 mg/kg body weight p.o. These were then treated as follows; Artesunate 2 mg/kg i.m (n = 5) in the form Artesun^®^ [37]. This dosage was chosen since 2 mg/kg was very safe in subchronic studies of Artesunate ranging from 2 to 10 mg/kg [38, 39]. Artesunate-Amodiaquine per os at 4/10 mg/kg p.o in the form Winthrop^®^ was used since this had previously been reported to have effects on gastric mucosa [40] while Artemether- Lumefantrine (2.3/27.4 mg/kg) from Combiart^®^ was administered as a follow up on a previously used dosage of artemether-lumefantrine (2/12 mg/kg) on gastric ulcers [40]. The positive control (with ulcers) was treated with normal saline at 1 ml/kg p.o. Furthermore, group five was the negative control (no ulcers) and this also received normal saline at 1 ml/kg p.o. All antimalarial agents and chemicals were procured from a licensed pharmacy in Ishaka town of Ishaka-Bushenyi municipality, Bushenyi, Uganda.

#### Determination of gastric ulcer index

The gastric ulcer index was determined 24 h after treatment, using standard methods [36]. The rats were euthanized using thiopental sodium since this is ethically acceptable in experimental animals [41] and stomachs were harvested through a *linea alba* incision. The stomach was immediately opened along the greater curvature, mucosa cleaned of any debris with normal saline and pinned wide onto a wax board for ulcer counting and length taking. The ulcer counting was done using a magnifying glass (×10). Any black-red spot or line along the longitudinal axis, on the mucosa, was counted as an ulcer. The length of each counted ulcer was taken with a divider and ruler and recorded. The sum of the ulcer-lengths was recorded as the ulcer index for the particular stomach (one black/red mucosal spot was considered to be 0.5 mm). For accuracy, the average of two counting and length takings was considered for each stomach.

#### Determination of gastric mucosa reduced glutathione

Gastric mucosa reduced glutathione as a marker of anti-oxidative activity was determined by the method as described previously [42]. 1 g of gastric mucosa scrapings was obtained, homogenized and then the supernatant was obtained after centrifugation at 3000 rpm, 40 °C for 10 min. The supernatant was reacted with 5, 5′-dithiol-bis-2 nitrobenzoic acid. Colorimetry was then carried out to obtain absorbance at 520 nm. The absorbance was compared with the standard curve to obtain the quantity of reduced glutathione, expressed as µm/g of mucosal tissue.

#### Determination of gastric mucosal malondialdehyde

Gastric mucosal malondialdehyde (MDA) was determined as a marker of lipid peroxidation, by the method as described previously [43]. 1 g of gastric mucosal scrapings was obtained and suspended in 20 ml of butylated hydroxytoluene (0.5 M) to avoid oxidation. The sample was homogenized in Tris–HCl (20 mM) for 15 s, then centrifuged at 3000 rpm, 4 °C for 10 min to obtain a supernatant. The supernatant was then reacted with N-methyl-2-phenylindole at 45 °C. The absorbance of the solution was taken with a colorimeter (Colorimeter 254 Sherwood^®^) at 540 nm. The quantity of MDA in the weighed mucosa scrapings was obtained by comparison of the spectrophotometer reading with the standard curve. The MDA was expressed as µmoles/g of tissue.

#### Statistical analysis

The data was recorded and then entered in MS Excel version 10 for statistical analysis. Descriptive statistics were conducted to determine the homogeneity of the data on ulcer index, reduced glutathione and malondialdehyde concentrations. Data was subjected to One way ANOVA with Tukey post hoc test and information was expressed as mean ± SD and presented on graphs and a Table, while significance (P < 0.05) was reported with different superscripts (a, b, c).

### Results

#### Effects of Artesunate-amodiaquine treatment on gastric ulcer index, oxidative and antioxidant status

**Fig. 1:**
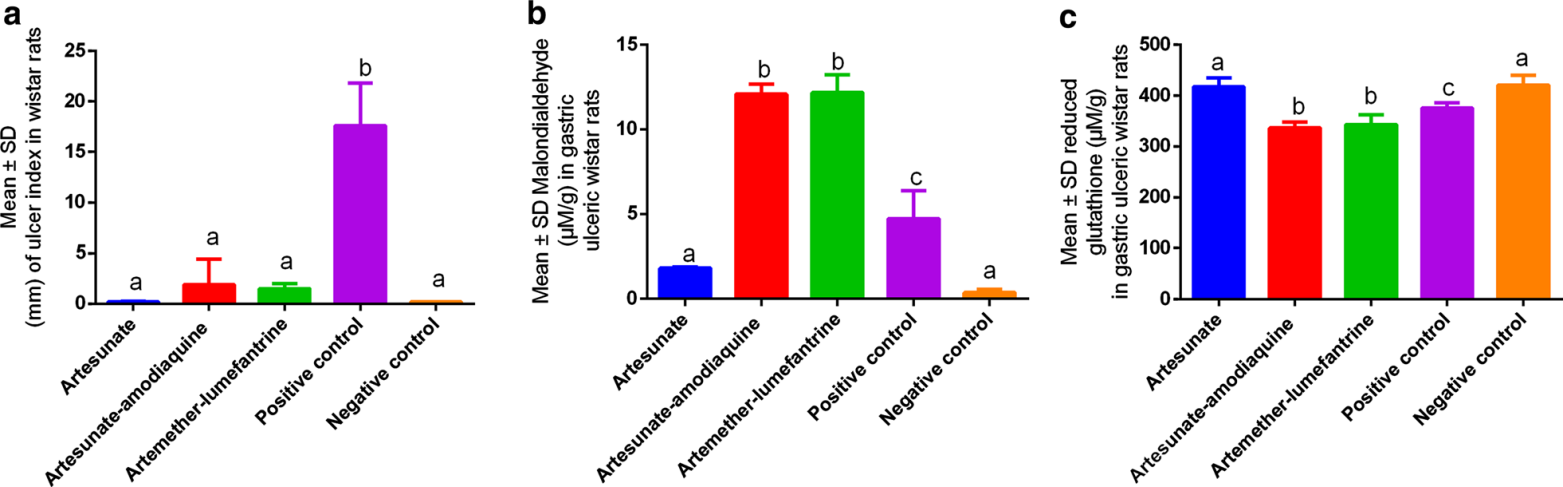
Variations in ulcer index, malondialdehyde and reduced glutathione in the gastric mucosa of male Wistar rats. Graphs **a** Ulcer index, **b** concentrations of malondialdehyde and **c** concentrations of reduced glutathione

The study showed that the ulcer index was relatively the same in all experimental animals except in the positive control (P < 0.05). Ulcer index was higher in the Artesunate-amodiaquine than Artemether-lumefantrine groups although no significant differences were observed (Fig. 1a). Malondialdehyde (MDA) levels were highest in the combination groups (P > 0.05) with significantly high concentrations observed in the antimalarial combinations and Artesunate (Fig. 1b). In addition, MDA levels were lower in the negative control and no significant differences were observed with Artesunate (P > 0.05). Furthermore, levels of reduced glutathione were significantly the same (P > 0.05) in the Artesunate and the negative control (Fig. 1c). Significantly (P < 0.05) low concentrations were associated with the combined therapies of antimalarial agents with both Artesunate and the Negative control as shown in Table 1.

**Table 1:** Multiple comparisons on ulcer index, malondialdehyde, and reduced glutathione in male Wistar rats for against each experimental group

Tukey’s multiple comparisons tests	Ulcer index	Malondialdehyde	Reduced glutathione
	Adjusted P values		
Artesunate vs. Artesunate-amodiaquine	0.6887	< 0.0001	< 0.0001
Artesunate vs. Artemether-lumefantrine	0.8519	< 0.0001	< 0.0001
Artesunate vs. positive control	< 0.0001	< 0.0003	0.0166
Artesunate vs. negative control	> 0.9999	0.0685	0.9973
Artesunate-amodiaquine vs. Artemether-lumefantrine	0.9982	0.9997	0.9701
Artesunate-amodiaquine vs. positive control	< 0.0001	< 0.0001	0.0348
Artesunate-amodiaquine vs. negative control	0.7211	< 0.0001	< 0.0001
Artemether-lumefantrine vs. positive control	< 0.0001	< 0.0001	0.1009
Artemether-lumefantrine vs. negative control	0.8704	< 0.0001	< 0.0001
Positive control vs. negative control	< 0.0001	< 0.0001	0.0118

#### Gastric mucosa histopathological lesions

**Fig. 2:**
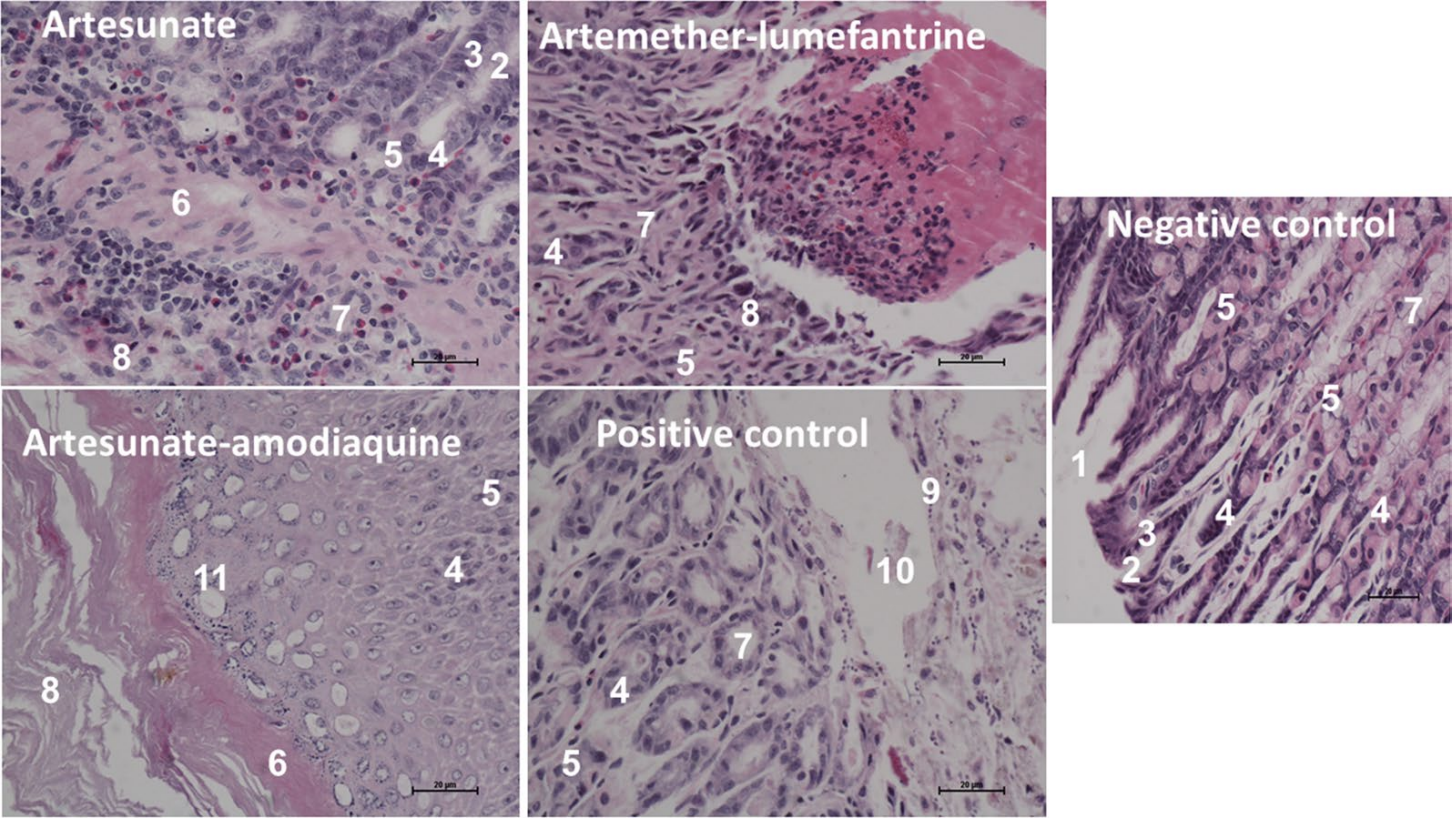
Histological changes in gastric mucosa and epithelia following administration of common antimalarials in male Wister rats. 1 = Gastric lumen; 2 = Gastric pit; 3 = Columnar epithelium; 4 = Parietal cells; 5 = Chief cells; 6 = Lamina muscularis; 7 = Gastric glands; 8 = Sub mucosa; 9 = Endothelium of blood vessel; 10 = Blood cell; 11 = Vacoulations in nonglandular stomach

The macroscopic analysis showed erosion of the gastric mucosa (ulcer index) while microscopic analysis demonstrated mild inflammation in Artesunate with infiltration by inflammatory cells. Combination therapies of antimalarials i.e. Artesunate-amodiaquine and Artemether-lumefantrine were associated with diffuse vacuolations in the non-glandular stomach and acute inflammation in the glandular stomach showing that pathological lesions are widespread in the gastric mucosa. Furthermore, the positive control was associated with severe basophilic bodies and debris in the mucosa and no lesions were found in the negative control as shown in Fig. 2.

## Discussion

Antimalarials were able to induce gastric ulcers following indomethacin administration and this was in agreement with previous studies [14, 20, 44]. The effects of antimalarials such as amodiaquine, quinine and chloroquine on gastric ulcer has been previously reported [3, 45]. Antimalarial combination therapies (ACTs) of Artesunate-amodiaquine and Artemether-lumefantrine (Fig. 1a) showed higher gastric ulcer index than Artesunate alone. These findings raise major therapeutical challenges on the safety of ACTs due to their ability to damage the gastric mucosa and mucous layer [14, 15]. The study showed that ACTs were associated with high levels of malondialdehyde (MDA) and low concentrations of reduced glutathione (Fig. 1b, c). This showed that ACTs exert their toxic effects through an increase of oxidative stress in body tissues thus upsetting the delicate oxidative-antioxidant status responsibly for the maintenance of the integrity of cell membranes leading to mucosal ulceration [18–22]. Findings in this study on AMTs are contrary to single ACTs such as Artesunate which was observed to have protective effects on the gastric mucosa (Fig. 1). Findings in this study demonstrate the safety of Artesunate and this was in agreement with previous studies [8, 9, 40]. This offers a firm basis for their safety [6]. In this study, the use of ACTs was found not to be safe, thus raising major global implications since ACTs are commonly used in the management of uncomplicated malaria [10].

Microscopically, Artesunate was found to be safer than ACTs (Fig. 2) showing that drugs including antimalarials are among the exogenous substances known to affect the gastric mucosa through oxidative stress mechanisms [12, 13, 17]. In addition, ACTs severe pro-oxidative stress properties stimulated vacuolations and severe inflammation. For example, Lumefantrine is a potent tissue pro-oxidant [27, 28] and it has been incorporated with Artemether into a combination therapy for the treatment of malaria [29–32]. The basic findings of this study demonstrate that ACTs might not be safe on the gastric mucosa. Furthermore, Artesunate-amodiaquine has been associated with gastrointestinal complications like vomiting, diarrhea and abdominal pain [33, 34], demonstrating that the Amodiaquine combination in the drug makes Artesunate lose its gastric protective effects (Figs. 1, 2). These findings support previous findings in which aminoquinoline derivatives including Amodiaquine have been used with reports of varying adverse effects including toxicity concerns and increasing frequency of development of drug resistance [3, 4] and are aggressive to the gastric mucosa precipitating gastric ulceration [3, 5].

## Limitations

The study investigated MDA-GSH axis thus to gain more information on the oxidative-antioxidant status, studies on more markers in oxidation, prostaglandins, disruption of local mucosal defense mechanisms, mucosal perfusion gastric mucus and bicarbonate secretion as well as inflammatory cytokines and molecular markers would help offer a more conclusive picture on antimalarial combination therapies.

## Data Availability

Data used in the study can be accessed at https://figshare.com/s/5bc742d01e28c4e23cc9.

## Abbreviations

ACTs: Antimalarial combination therapies
AMTs: Antimalarial single therapies
GSH: Reduced glutathione
HCl: Hydrochloric acid
i.m: Intramuscular
MDA: Malondialdehyde
mg/kg: Milgram per kilogram
p.o: Per os
WHO: World Health Organisation
