# Correction to: Antimalarial combination therapies increase gastric ulcers through an imbalance of basic antioxidative-oxidative enzymes in male Wistar rats

**DOI:** 10.1186/s13104-020-05084-4

**Published:** 2020-05-18

**Authors:** Muhamudu Kalange, Miriam Nansunga, Keneth Iceland Kasozi, Josephine Kasolo, Jackline Namulema, Jovial Kasande Atusiimirwe, Emmanuel Tiyo Ayikobua, Fred Ssempijja, Edson Ireeta Munanura, Kevin Matama, Ibrahim Semuyaba, Gerald Zirintunda, Alfred O. Okpanachi

**Affiliations:** 1 Department of Physiology, Faculty of Biomedical Sciences, Kampala International University, Western Campus, Box 71, Bushenyi, Uganda; 2 Department of Physiology, College of Health Sciences, Makerere University, Box 7062, Kampala, Uganda; 3 Department of Physiology, Faculty of Biomedical Sciences, School of Medicine, Soroti University, Soroti, Uganda; 4 Department of Anatomy, Faculty of Biomedical Sciences, Kampala International University, Western Campus, Box 71, Bushenyi, Uganda; 5 Department of Pharmacy, College of Health Sciences, Makerere University, Box 7062, Kampala, Uganda; 6 Department of Therapeutics and Toxicology, School of Pharmacy, Kampala International University Western Campus, Box 71, Bushenyi, Uganda; 7 Department of Animal Production, Faculty of Agriculture and Animal Sciences, Busitema University Arapai Campus, Box 203, Soroti, Uganda; 8 Department of Physiology, Faculty of Medicine, Uzima University College CUEA, Box 2502, Kisumu, Kenya; 9 Department of Physiology, Faculty of Health Sciences, Busitema University, Mbale, Uganda

## Correction to: BMC Res Notes (2020) 13:230 10.1186/s13104-020-05073-7

The original version of this article [1] unfortunately included an error to an author’s name. Author Emmanuel Tiyo Ayikobua was erroneously presented as Emanuel Tiyo Ayikobua.

The correct author name has been included in the author list of this Correction article and is already updated in the original article.
